# Overexpression of parkin protects retinal ganglion cells in experimental glaucoma

**DOI:** 10.1038/s41419-017-0146-9

**Published:** 2018-01-24

**Authors:** Yi Dai, Xinxin Hu, Xinghuai Sun

**Affiliations:** 1 Department of Ophthalmology and Vision Science, Eye & ENT Hospital, Shanghai Medical College, Fudan University, Shanghai, China; 2 Key Laboratory of Myopia of State Health Ministry and Key Laboratory of Visual Impairment and Restoration of Shanghai, Shanghai, 20031 China

## Abstract

Glaucoma is a leading cause of irreversible blindness and characterized by progressive damage of retinal ganglion cells (RGCs). Growing evidences have linked impaired mitophagy with neurodegenerative diseases, while the E3 ubiquitin ligase parkin may play a key role. However, the pathophysiological relationship between parkin and glaucoma remains largely unknown. Using chronic hypertensive glaucoma rats induced by translimbal laser photocoagulation, we show here that the protein level of parkin and its downstream optineurin proteins were increased in hypertensive retinas. The ratio of LC3-II to LC3-I, the number of mitophagosomes, and unhealthy mitochondria were increased in hypertensive optic nerves. Overexpression of parkin by viral vectors increased RGC survival in glaucomatous rats in vivo and under excitotoxicity in vitro. It also promoted optineurin expression and improved mitochondrial health. In parkin-overexpressed glaucomatous rats, the ratio of LC3-II to LC3-I, LAMP1 level, and the number of mitophagosomes in optic nerve were decreased at 3 days, yet increased at 2 weeks following intraocular pressure (IOP) elevation. These findings demonstrate that dysfunction of mitophagy exist in RGCs of glaucomatous rats. Overexpression of parkin exerted a significant protective effect on RGCs and partially restored dysfunction of mitophagy in response to cumulative IOP elevation.

## Introduction

Glaucoma, characterized by progressive damage of retinal ganglion cells (RGCs), is a neurodegenerative disease that leads to irreversible blindness^1^. The mechanism of RGC death in glaucoma is complicated and not fully understood. Intraocular pressure (IOP) elevation is considered to be the major risk factor for glaucoma and so far the only treatable factor^2^. However, lowering IOP is not always sufficient to prevent progression of glaucomatous optic neuropathy^3^. Recent studies have shown that impairment of mitochondrial dynamics may play a key role in glaucomatous RGC loss^4,5^. Modulation of mitochondrial fission- and fusion-related proteins, such as OPA1 and DRP1, have been found to protect both RGCs and their axons in experimental models of glaucoma and other optic neuropathies^6–8^.

Mitophagy is the selective degradation of mitochondria, in which the E3 ubiquitin ligase parkin targets damaged mitochondria for degradation by autophagosomes^9^. When the mitochondrial membrane potential is depolarized, parkin is recruited to the outer mitochondrial membrane, leading to the parkin-mediated ubiquitination of mitochondrial membrane proteins and facilitating mitophagy^10^. Parkin promotes recruitment of optineurin, an autophagy receptor^11^, which is actively recruited to ubiquitinated mitochondria downstream of parkin. Growing evidences have linked impaired mitophagy with neurodegenerative diseases, such as Parkinson’s and Alzheimer’s diseases^12,13^. Mutations in parkin by impairing mitophagy were found in patients with Parkinson’s disease^13^ and in experimental models^14^. Meanwhile, parkin is widely accepted to be a potent neuroprotectant involved in multiple pathways^15^. Our previous study has demonstrated that parkin overexpression exerted a significant protective effect on cultured RGCs against glutamate excitotoxicity^16^. However, the pathophysiological relationship between parkin and glaucoma remains largely unknown.

Therefore, the aim of present study was undertaken to investigate the role of parkin in regulating mitophagy and whether overexpression of parkin can protect RGC in experimental glaucoma.

## Results

### IOP elevation, change of RGC survival, and protein expression in chronic hypertensive glaucoma rats

All laser-treated eyes had significantly elevated IOP compared with their contralateral control eyes (Table 1).

**Table 1:** IOP exposure in the experimental glaucoma and control eyes

Laser treated	Mean IOP (mm Hg)		Mean IOP (mm Hg)	
Time (*n*)	Glaucomatous	Control	AAV2-parkin	AAV2-null
1 Day	32.1 ± 10.5** (*n* = 29)	10.7 ± 1.2 (*n* = 29)	31.4 ± 12.0** (*n* = 23)	30.0 ± 11.3** (*n* = 23)
3 Days	22.2 ± 6.8** (*n* = 29)	9.8 ± 0.9 (*n* = 29)	21.5 ± 5.7** (*n* = 23)	22.9 ± 8.1** (*n* = 23)
1 Week	18.9 ± 4.5** (*n* = 21)	10.2 ± 1.2 (*n* = 21)	19.3 ± 5.3** (*n* = 21)	19.7 ± 5.7** (*n* = 21)
2 Weeks	17.7 ± 2.7** (*n* = 12)	11.0 ± 1.0 (*n* = 12)	18.4 ± 4.2** (*n* = 12)	19.6 ± 4.4** (*n* = 12)

Mean IOP is the average IOP after induction of experimental glaucoma

**P < 0.01 compared with control eyes

**Fig. 1:**
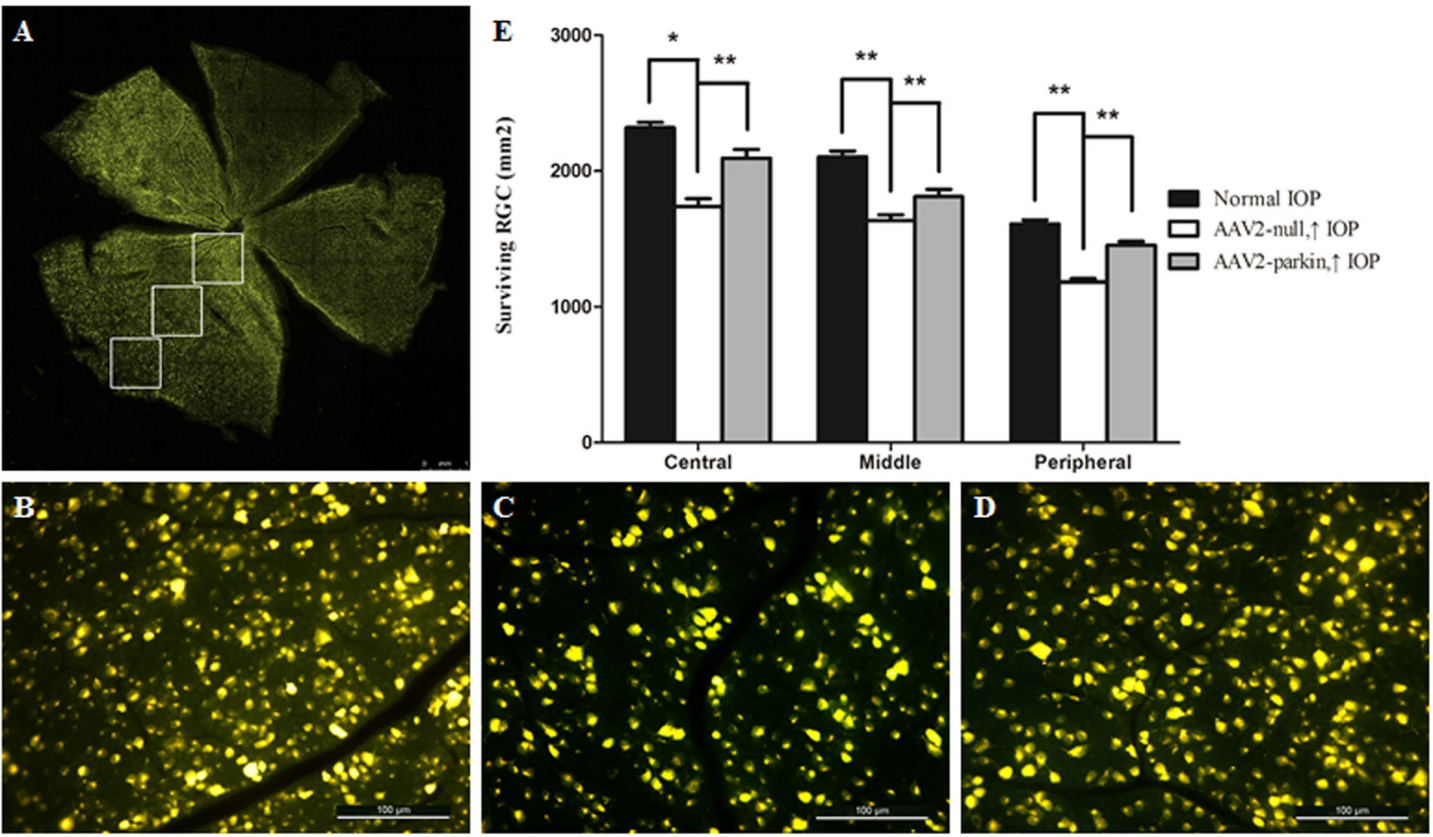
Retinal ganglion cell (RGC) survival in the hypertensive rat retina after overexpression of parkin The retinal flat mounts of AAV2-parkin-transfected hypertensive rat **a**, **d**, control rat **b**, and AAV2-null-transfected hypertensive rat **c** The quantitative analysis of RGC survival e. *n* = 6, data are expressed as mean ± SD, *P < 0.05, **P < 0.01. Scale bar = 1 mm. **a** Scale bar = 100 μm **b**–**d**

**Fig. 2:**
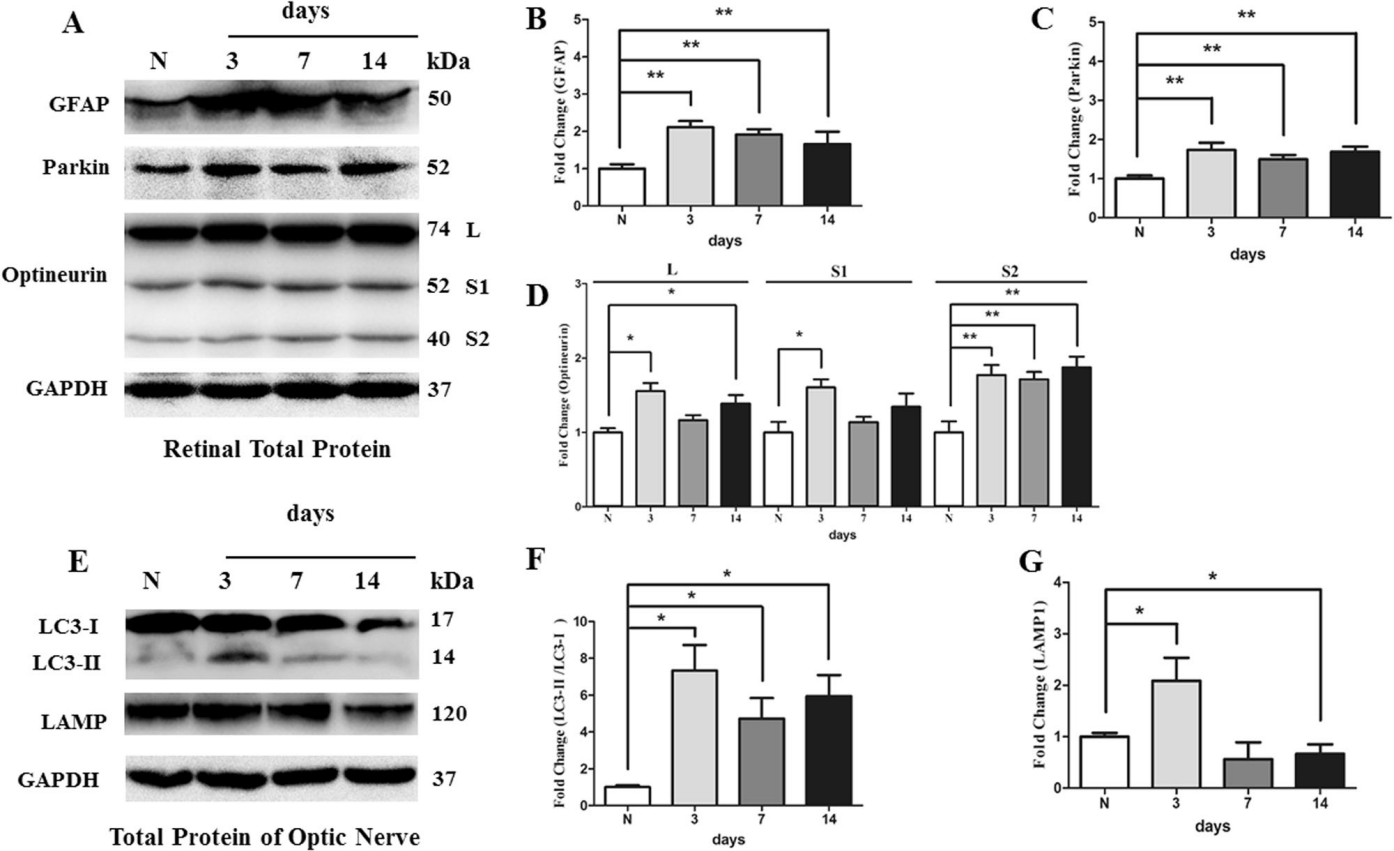
Western blot analysis of the GFAP, parkin, optineurin, LC3-I and -II, and LAMP1 proteins in hypertensive retina and optic nerve Compared with control retina, increased expression of the GFAP protein **b**, parkin protein **c**, and optineurin protein **d** were observed at 3 days, 1 week, and 2 weeks in hypertensive retinas. The ratio of LC3-II to LC3-I was significantly increased at 3 days, 1 week, and 2 weeks in hypertensive optic nerves f, and the expression of LAMP1 protein was increased at 3 days, yet decreased at 2 weeks in hypertensive optic nerves g. *n* = 4, data are expressed as mean ± SD, *P < 0.05 compared with control retina. **P < 0.01 compared with control retina

The normal rat retina had an average of 2320 RGCs/mm^2^ in the central, 2104 RGCs/mm^2^ in the middle, and 1610 RGCs/mm^2^ in the peripheral areas (*n* = 6 retinas; Fig. 2b). Compared with the contralateral control eyes, laser-treated eyes had a 25% RGC loss in the central, 22% in the middle, and a 27% in the peripheral retina (Fig. 1c) 2 weeks after IOP elevation (*n* = 6 retinas, *P* < 0.01; Fig. 1e).

After IOP elevation, the immunoreactivity of glial fibrillary acidic protein (GFAP) was increased in the Muller cells in hypertensive retinas compared with contralateral control eyes (Fig. 4e, f). Western blot analysis showed that the protein level of GFAP was significantly increased at 3 days, 1 week, and 2 weeks in hypertensive retinas (*P* < 0.01; Fig. 2a, b).

**Fig. 3:**
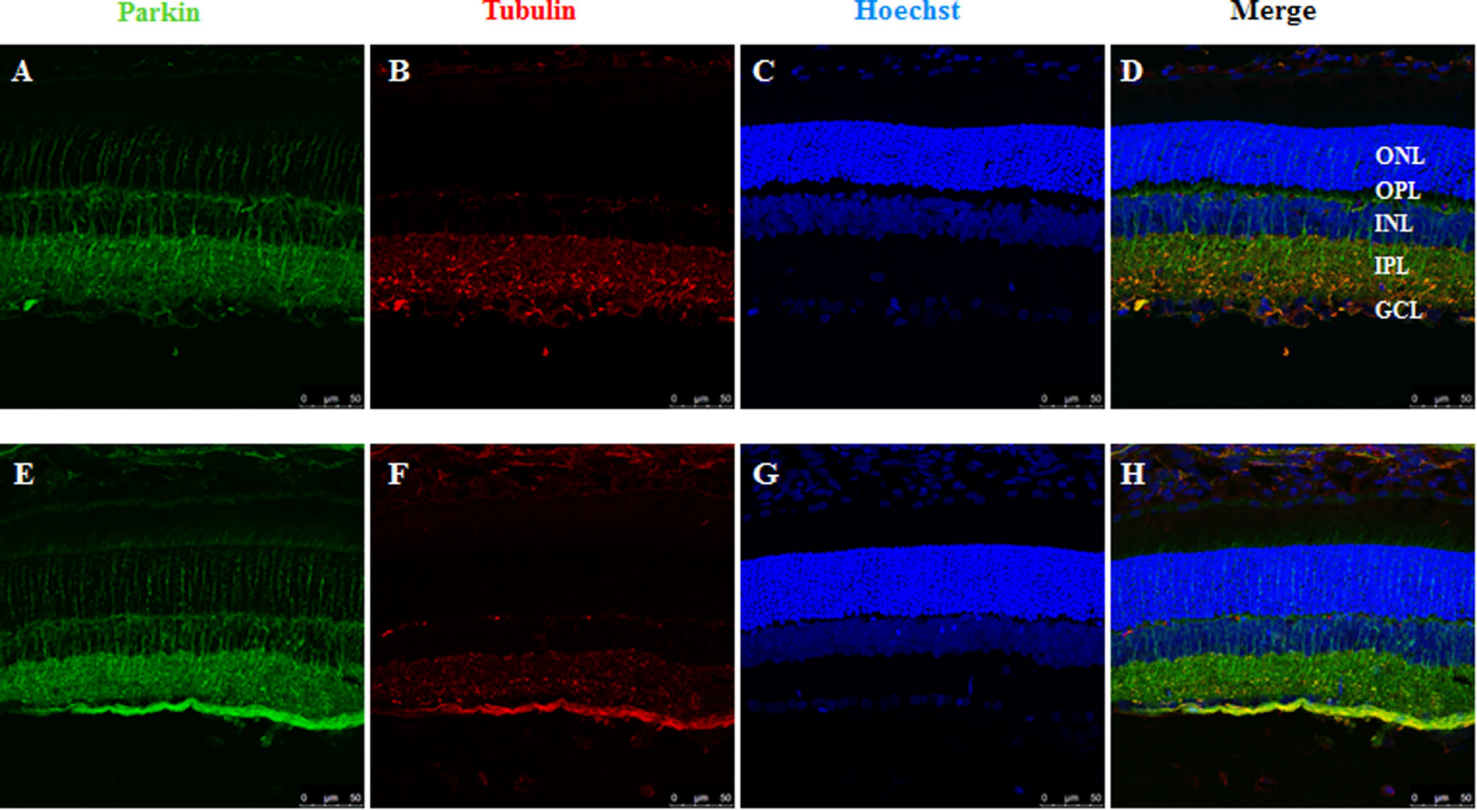
Immunofluorescence analysis of parkin in control and hypertensive retinas Parkin protein expression was co-localized with Tubulin, a marker of retinal ganglion cells. Compared with control retina **a**–**d**, parkin immunoreactivity was greater, especially in retinal nerve fiber layer of hypertensive retinas. **e**–**h** Scale bar = 25 μm

Mitophagy-related parkin and optineurin protein expression levels were co-localized with Tubulin, a marker of RGCs, as shown in the rat retina (Fig. 3a–d). Compared with the control groups, the immunoreactivity of parkin and optineurin was increased in the ganglion cell layer of hypertensive retina (Fig. 3e–h). Western blot analysis further showed that the protein level of parkin was significantly upregulated at 3 days, 1 week, and 2 weeks in the hypertensive retinas after IOP elevation (*P* < 0.01, Fig. 2a, c). In addition, the level of isoform L of optineurin protein were increased at 3 days and 2 weeks in the hypertensive retinas. The level of isoform S1 of optineurin protein were increased at 3 days in the hypertensive retinas. And increases in the level of isoform S2 of optineurin protein were observed at 3 days, 1 week, and 2 weeks in the hypertensive retinas (*P* < 0.05, Fig. 2a, d).

### Mitochondrial morphology and mitophagy in RGC of chronic hypertensive glaucoma rats

Ultrastructural studies showed that the number of mitochondria was significantly increased in hypertensive optic nerve at 3 days and 2 weeks (Fig. 5d). Mitochondria was in close proximity to each other, indicating the scene of fusion or fission events (Fig. 5i). A mitochondrial health scale (Fig. 5f) was presented based primarily on cristae appearance, with four representing mitochondria of the healthiest appearance. Hypertensive optic nerves at 3 days and 2 weeks have significantly poorer health scores than those in controls (Fig. 5e).

Compared with the control group, autophagosomes and mitophagosomes were apparently more in hypertensive optic nerve at 3 days and 2 weeks (Fig. 5g, h). The ratio of LC3-II to LC3-I was significantly increased at 3 days, 1 week, and 2 weeks in hypertensive optic nerves (*P* < 0.05, Fig. 2e, f). With regard to LAMP1, increases in this protein were observed at 3 days, yet decreases at 2 weeks in hypertensive optic nerves (*P* < 0.05, Fig. 2e, g).

### Effect of parkin overexpression on RGC survival and Muller glial activation

Rats in the AAV2-parkin-transfected groups and AAV2-null-transfected groups underwent translimbal laser-induced IOP elevation 3 weeks after injection of AAV2 vectors. All laser-treated eyes had significantly elevated IOP (Table 1). There were no significant differences in mean or peak IOP between the AAV2-parkin and AAV2-null groups.

**Fig. 4:**
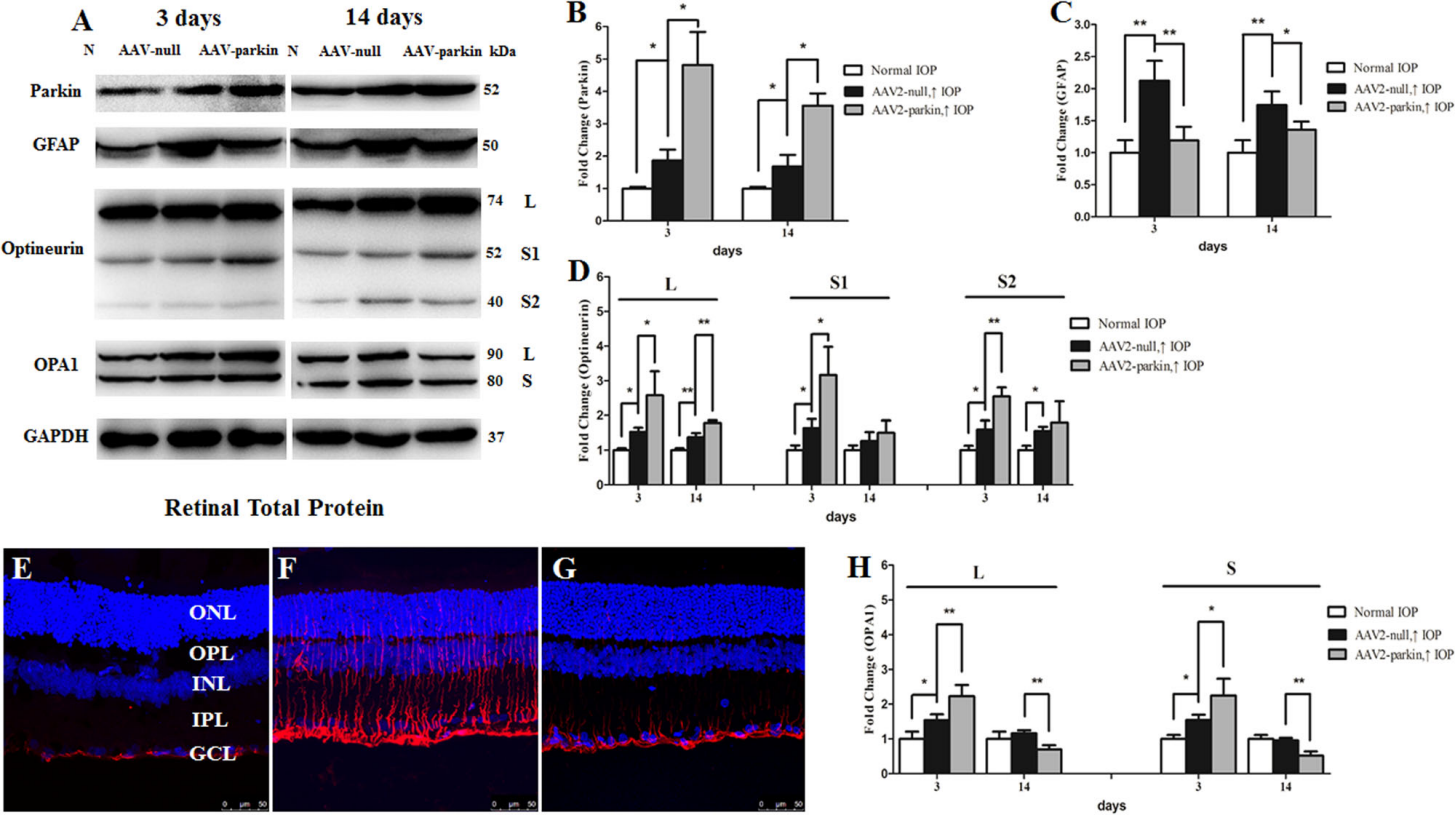
Effect of parkin overexpression on hypertensive retina Western blot analysis of parkin, GFAP, OPA1, and optineurin proteins in parkin-transfected retina. **a**–**d**, **h** Compared with AAV2-null-transfected retinas, parkin expression in AAV2-parkin-transfected retinas was significantly increased at 3 days and 2 weeks **a**, **b**, and the protein level of GFAP was decreased. **a**, **c** Expression of isoform L of optineurin protein in AAV2-parkin-transfected hypertensive retinas was increased at 3 days and 2 weeks, and isoform S1 and S2 of optineurin protein was increased at 3 days. **a**, **d** Expression of isoform L and S of the OPA1 protein in the AAV2-parkin-transfected hypertensive retinas was increased at 3 days, yet decreased at 2 weeks. **a**, **h** Compared with the control retina **e**, GFAP immunoreactivity was induced in the Muller cells of the AAV2-null-transfected hypertensive retina. **f** Overexpression of parkin decreased GFAP immunoreactivity in Muller cells **g** of the hypertensive retina. *n* = 4, data are expressed as mean ± SD, *P < 0.05. **P < 0.01. Scale bar = 50 μm **e**–**g**

The efficiency of transgene expression was measured by detecting the levels of parkin with immunoblotting in retinas. Parkin expression was significantly increased in the AAV2-parkin-transfected groups compared with that in the AAV2-null-transfected groups at 3 days, 1 week, and 2 weeks in the hypertensive retinas (*P* < 0.01, Fig. 4a, b).

Overexpression of parkin significantly increased RGC survival by 19% in the central retina, by 13% in the middle retina, and by 23% in the peripheral retina compared with those transfected with AAV2 null in hypertensive rats (*n* = 6 retinas, *P* < 0.01; Fig. 1d, e).

Western blot and immunofluorescence analysis showed that the protein level of GFAP was decreased in AAV2-parkin-transfected hypertensive retinas at 3 days and 2 weeks (*P* < 0.05; Fig. 4a, c, f, g). Moreover, the level of isoforms L and S of the OPA1 protein was increased in the AAV2-parkin-transfected hypertensive retinas at 3 days, yet decreased at 2 weeks (*P* < 0.05; Fig. 4a, h).

### Impacts of parkin overexpression on mitophagy in chronic hypertensive glaucoma rats

Western blot analysis demonstrated that, compared with the AAV2-null-transfected groups, the level of isoform L of optineurin protein was increased in AAV2-parkin-transfected hypertensive retinas at 3 days and 2 weeks. And the level of isoforms S1 and S2 of optineurin protein was increased at 3 days in the AAV2-parkin-transfected hypertensive retinas (*P* < 0.05; Fig. 4a, d).

**Fig. 5:**
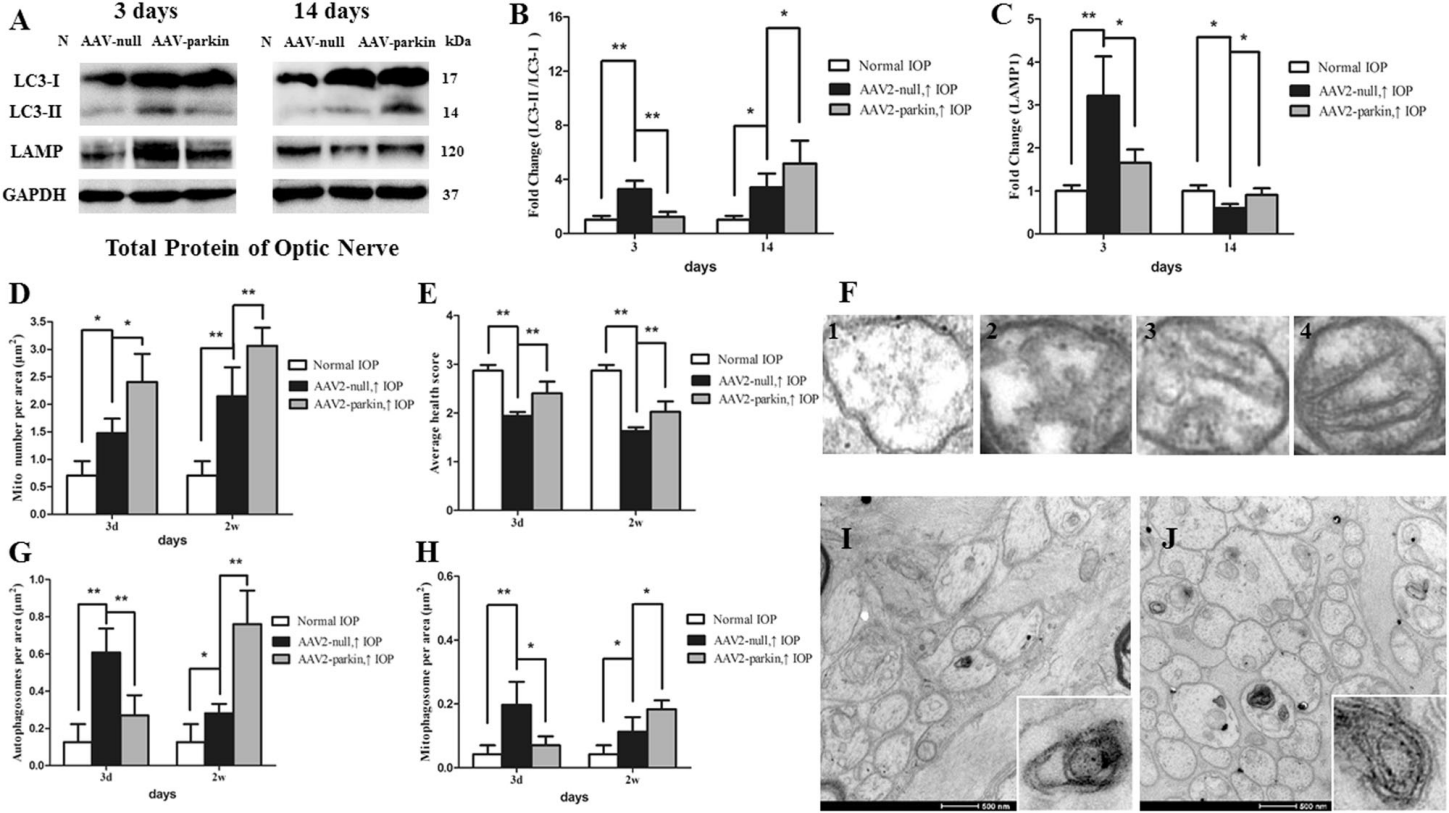
Impacts of parkin overexpression on mitophagy in hypertensive optic nerve Western blot analysis of LC3-I and -II and LAMP1 proteins in parkin-transfected optic nerve. Compared with AAV2-null-transfected groups, the ratio of LC3-II to LC3-I (**a**, **b**), and the expression of LAMP1 protein **a**, **c** in AAV2-parkin-transfected hypertensive optic nerves were decreased at 3 days, yet increased at 2 weeks. Ultrastructural analysis of mitochondrial morphology and mitophagy. **d**–**j** A mitochondrial health scale **f** was presented based primarily on cristae appearance, with four representing mitochondria of the healthiest appearance. Compared with control rats, the number of mitochondria **d**, autophagosomes **g**, and mitophagosomes **h** were significantly increased in hypertensive optic nerve at 3 days and 2 weeks, while mitochondrial health scales were decreased. **e** Compared with the AAV2-null-transfected groups, higher mitochondrial health scores **e** and more number of mitochondria **d** were observed at 3 days and 2 weeks **i**, **j** in AAV2-parkin-transfected groups. The number of autophagosomes **g** and mitophagosomes **h** was significantly decreased at 3 days, while increased at 2 weeks. **i**, **j** *n* = 3, data are expressed as mean ± SD, *P < 0.05. **P < 0.01. Scale bar = 500 nm **i**–**j**

Moreover, when compared to the AAV2-null-transfected groups, the ratio of LC3-II to LC3-I were lower at 3 days in AAV2-parkin-transfected hypertensive optic nerves, yet was higher at 2 weeks after IOP elevation (*P* < 0.05; Fig. 5a, b). The protein level of LAMP1 was decreased at 3 days in AAV2-parkin-transfected hypertensive optic nerves, yet was increased at 2 weeks after IOP elevation (*P* < 0.05; Fig. 5a, c).

In comparison with the AAV2-null-transfected groups, representative images from transmission electron microscopy in the AAV2-parkin-transfected groups showed higher mitochondrial health scores and contained more number of mitochondria at 3 days and 2 weeks (*P* < 0.01; Fig. 5e, d). Consistent with the ratio of LC3-II to LC3-I in western blot analysis, quantitative analysis showed that the number of autophagosomes and mitophagosomes was significantly decreased at 3 days, while increased at 2 weeks in AAV2-parkin -transfected groups (*P* < 0.01; Fig. 5g–j).

### Effects of parkin overexpression on RGCs under glutamate excitotoxicity

**Fig. 6:**
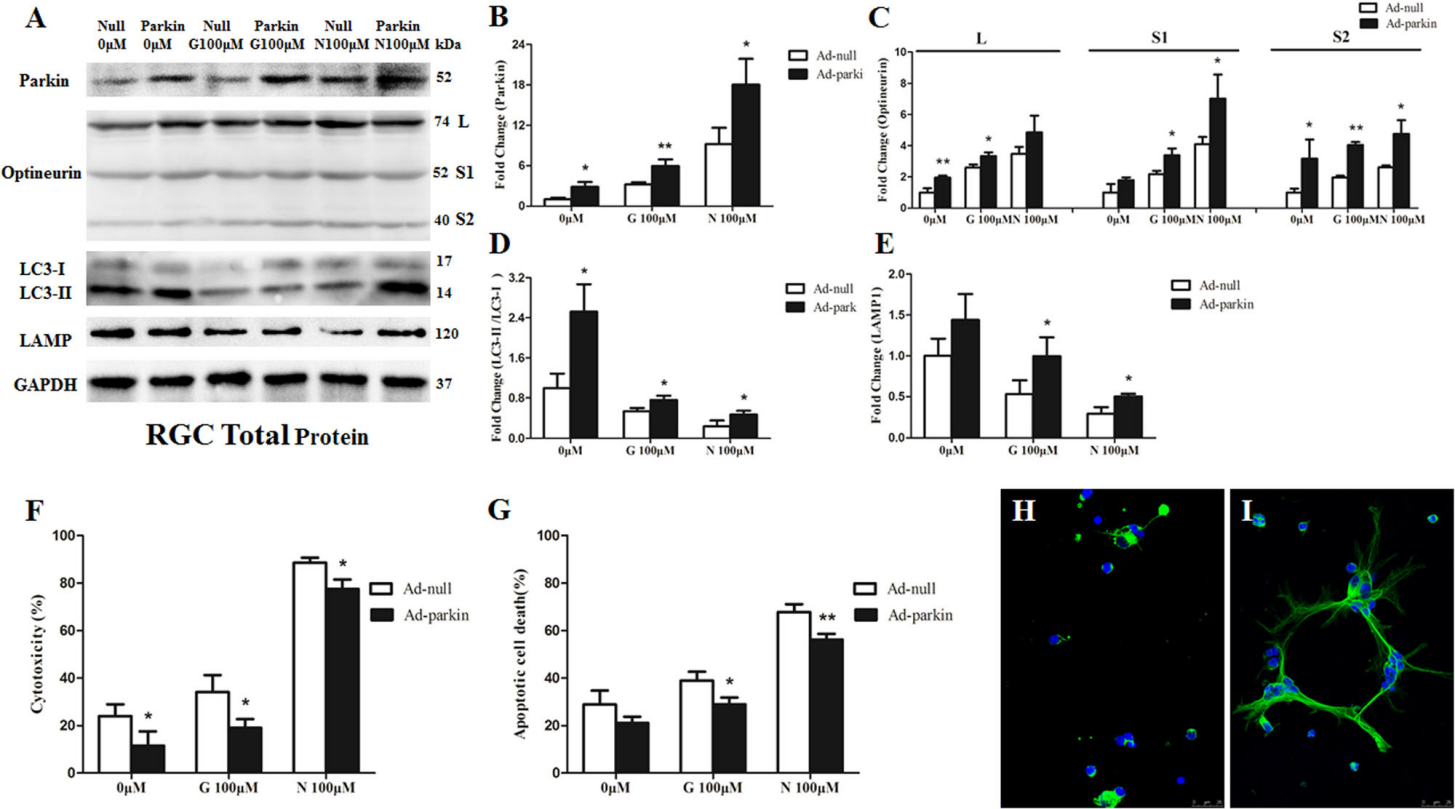
Effects of parkin overexpression on retinal ganglion cells (RGCs) under glutamate excitotoxicity Compared with Ad-null-transfected RGCs, the expression of parkin protein **a**, **b** and immunoreactivity of parkin **f**, **g** were upregulated in Ad-parkin-transfected RGCs. Expression of isoform S2 of the optineurin protein in Ad-parkin-transfected RGCs was increased in the 0 and 100 μM glutamate and 100 μM NMDA groups, expression of isoform L of the optineurin protein was increased in the 0 and 100 μM glutamate groups, and expression of isoform S1 of the optineurin protein was increased in the 100 μM glutamate and 100 μM NMDA groups **c**. The ratio of LC3-II to LC3-I was increased in the 0 and 100 μM glutamate and 100 μM NMDA groups. **d** Expression of LAMP1 protein was increased in the 100 μM glutamate and 100 μM NMDA groups. **e** The Ad-parkin-transfected RGCs showed lower level of cytotoxicity **f** and less apoptotic cell death **g**–**i** under glutamate excitotoxicity. **c** *n* = 3, data are expressed as mean ± SD, *P < 0.05 compared with the Ad-null-transfected groups, **P < 0.01 compared with the Ad-null-transfected groups. Scale bar = 25 μm **f**, **g**

The expression of parkin protein (Fig. 6a, b) and immunoreactivity of parkin (Fig. 6h, i) were upregulated in RGCs transfected with Ad-parkin (*P* < 0.01). Compared with the Ad-null-transfected RGCs, the cytotoxicity of Ad-parkin-transfected RGCs cultured in neurobasal medium was decreased (*P* < 0.05, Fig. 6f), while the protein level of optineurin and the ratio of LC3-II to LC3-I was increased (*P* < 0.05, Fig. 6a, d). These results indicated that overexpression of parkin has a positive influence on RGC viability.

**Fig. 7:**
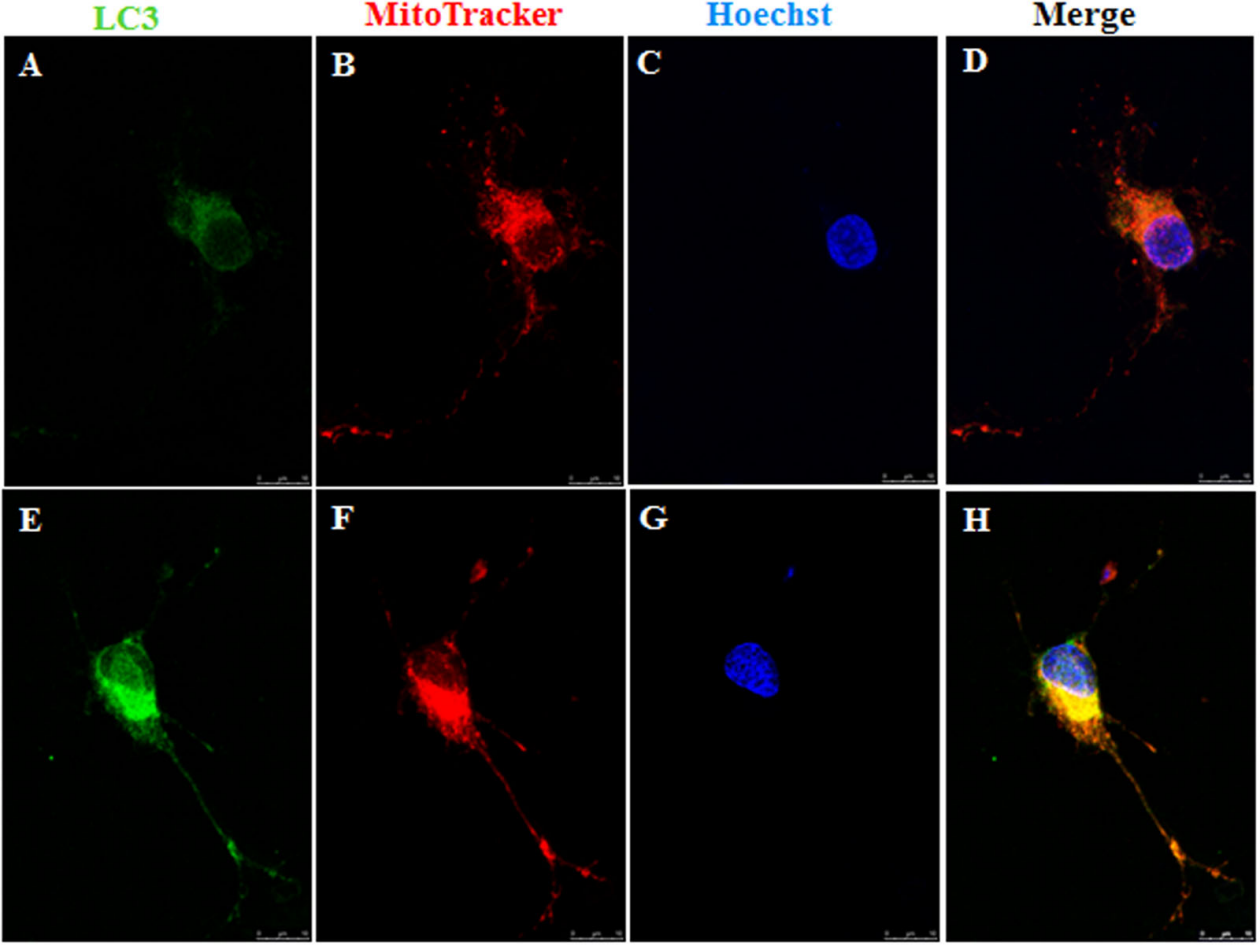
Retinal ganglion cells (RGCs) co-stained with MitoTracker Red and LC3 Glutamate treatment induced small spherical mitochondria and low immunoreactivity of LC3. **a**–**d** Overexpression of parkin increased the number of mitochondria, reduced glutamate-induced mitochondrial fragmentation, upregulated the immunoreactivity of LC3, and the co-localization between mitochondria and LC3 in the axons of the RGCs **e**–**h**

With regard to 100 μM glutamate and 100 μM *N*-methyl-d-aspartate (NMDA) treatment to RGCs, it induced 9.7 and 43.2% of apoptotic cells, respectively. Compared with those transfected with Ad null in RGCs, overexpression of parkin significantly decreased apoptotic cell by 25.6% under glutamate treatment and 17.1% under NMDA treatment (Fig. 6g–i). Lactate dehydrogenase (LDH) measurements also showed that overexpression of parkin significantly decreased cytotoxicity (*P* < 0.05, Fig. 6f) in RGCs under excitotoxicity. Western blot analysis showed that overexpression of parkin promoted the protein level of optineurin, LAMP1, and the ratio of LC3-II to LC3-I in glutamate and NMDA treatments (*P* < 0.05, Fig. 6a, c–e). Moreover, overexpression of parkin increased the number of mitochondria and reduced glutamate-induced mitochondrial fragmentation in the axons of the RGCs. It also upregulated the immunoreactivity of LC3 and the co-localization between mitochondria and LC3 in the axons of the RGCs (Fig. 7a–h).

## Discussion

These results demonstrate that the number of unhealthy mitochondria and mitophagosomes was increased in hypertensive optic nerve. Overexpression of parkin protected against RGC loss, attenuated GFAP expression, promoted optineurin expression, improved mitochondrial health, and partially restored dysfunction of mitophagy in chronic hypertensive glaucoma rats.

Selective autophagy of mitochondria, known as mitophagy, is an important mitochondrial quality-control mechanism^12^. Parkin is an E3 ubiquitin ligase that is expressed in multiple tissues. When the mitochondrial membrane potential is depolarized, parkin is recruited to the outer mitochondrial membrane, leading to the parkin-mediated ubiquitination of mitochondrial membrane proteins and facilitating mitophagy^17^. In this study, parkin expression was upregulated in RGC layer of hypertensive retina. Optineurin, an autophagy receptor, which is recruited to ubiquitinated mitochondria downstream of parkin^11^, was also upregulated in RGC layer of hypertensive retina. Ubiquitinated mitochondria with optineurin could then recruit the phagophore protein LC3, resulting in the formation of mitophagosomes and ultimate degradation of mitochondria via lysosome^18^. LC3, a marker of autophagosomes, exists in two forms. When autophagy is induced, cytosolic LC3-I is converted to the lipadated form, LC3-II, which associates with both the outer and inner membranes of the autophagosome^19^. Increasing level of LC3-II to LC3-I ratio has been used to monitor the induction of autophagy^20^. Our study found that the ratio of LC3-II to LC3-I and the number of mitophagsome were significantly increased in hypertensive optic nerve. In order to evaluate whether the increase in the ratio of LC3-II to LC3-I was caused by a reduction in lysosomal activity or increased autophagic flux, we investigated the expression of lysosomal marker, LAMP1. The result showed that LAMP1 was increased at 3 days following IOP elevation in hypertensive optic nerve, yet it was decreased at 2 week groups. The increased ratio of LC3-II to LC3-I and decreased LAMP1 may represent decreased autophagosome turnover and decreased opportunity for local mitochondrial recycling^20^ in optic nerve following sustained IOP elevation.

Growing evidence has implicated that mitophagy exerts a protective role in various pathological models via removing damaged mitochondria. Previous studies have shown the increased presence of autophagosomes^21–23^ and mitophagosomes^7^ in the RGCs of glaucoma models. Recently, Coughlin et al. reported that deteriorating mitochondria were not being efficiently recycled by mitophagy in glaucomatous DBA/2J mice^20^. Our data showed that mitophagosomes and the number of unhealthy mitochondria were increased in optic nerve of hypertensive rats. Taken together, it is plausible that mitophagy is increased to cope with stress in early neurodegenerative events (within 3 days) in hypertensive rats, yet with the cumulative damage of moderate IOP elevation, the compensatory mechanism begin to fail, and mitophagy is impaired along with the progressive RGC damage.

In order to understand the mechanism involved in parkin, mitophagy, and glaucoma, we studied whether overexpression of parkin can protect RGCs in chronic hypertensive glaucoma rats in vivo and under excitotoxicity in vitro. Our data showed that overexpression of parkin increased RGC survival both in vivo and in vitro. Previous studies have shown that OPA1, a dynamin-related GTPase that promotes mitochondria fusion, may mediate the protective effect of parkin in RGCs under moderate stress with minor mitochondrial dysfunction. In this study, OPA1 expression was upregulated at 3 days in parkin-overexpressed retina, while decreased at 2 weeks following IOP elevation. With regard to mitophagy pathway, overexpression of parkin induced downregulation of the ratio of LC3-II to LC3-I, LAMP1, and the number of mitophagosomes in hypertensive optic nerve 3 days following IOP elevation. These data suggest that parkin overexpression protected against RGC loss and may attenuate mitophagy in early neurodegenerative events (within 3 days) in hypertensive rats.

Emerging evidences have shown that overexpression of parkin recovered an efficient mitophagic flux and potentiate basal mitophagy^24,25^. In the current study, overexpression of parkin induced a significant upregulation of optineurin protein both in vivo and in vitro. A significant upregulation of the ratio of LC3-II to LC3-I, LAMP1, and the number of mitophagsome were observed in optic nerve at 2 weeks following IOP elevation, while the status of mitochondrial health was improved. In vitro study also shown that overexpression of parkin upregulated the ratio of LC3-II to LC3-I and the level of LAMP1 in RGCs under glutamate excitotoxicity. Of interest, parkin overexpression was found to improve mitophagy and extend lifespan in *Caenorhabditis elegans*^15^. All these data support the assumption that, in line with a neuroprotective role of parkin to RGCs, overexpression of parkin may partially restore dysfunction of mitophagy via enhancing recycle of deteriorating mitochondria in response to cumulative stress of IOP elevation. Further studies are needed to investigate whether small-molecule-promoting parkin-mediated mitophagy, such as de-ubiquitylation enzyme inhibitors, could be potential therapeutic targets against glaucomatous neurodegeneration.

In summary, our findings demonstrate that dysfunction of mitophagy exist in RGCs of hypertensive rats. Overexpression of parkin exerted a significant protective effect on RGCs and partially restored dysfunction of mitophagy under cumulative stress of IOP elevation. Interventions to modulate the parkin-mediated mitochondria pathway may be useful in protecting RGCs in glaucoma.

## Materias and methods

### Animals

All procedures concerning animals were in accordance with the ARVO Statement for the Use of Animals in Ophthalmic and Vision Research and under protocols approved by the Animal Ethics Committee of the Eye and ENT Hospital of Fudan University, China. Male Sprague-Dawley rats (240–300 g in weight) were raised under a 12-h light/12-h dark cycle and with free access to water and standard rodent diet.

### Experimental glaucoma

Chronic hypertensive glaucoma model was induced by translimbal laser photocoagulation of the trabecular meshwork as previously described^5,26^. All rats were anesthetized intraperitoneally with a mixture of ketamine (80 mg/kg, Gutian Pharmaceutical Co., Ltd, Fujian, China) and xylazine (5 mg/kg, Sigma-Aldrich, St. Louis, MO). Rat eyes were also treated with 0.4% oxybuprocaine hydrochloride (Santen) drops. Approximately 60–100 trabecular burns distributed around the limbus were delivered to each rat by argon laser (532 nm wavelength, 300 mW power, 0.5-s duration, 50-mm diameter spot size, Coherent ULTIMA2000, USA).

Elevated IOP was monitored in each eye under anesthesia with a hand-held tonometer (TonoLab; Tiolat Oy, Helsinki, Finland). IOP was measured before laser treatment and 1, 3, and 7 days after each treatment. At each time measuring IOP, slit-lamp ophthalmoscopy was used to exam the rat eyes. If anterior chamber inflammation or hemorrhage was observed, the rat was excluded from further experiment. The laser treatment was repeated after 1 week for all rats except those killed at 3 and 7 days.

### Plasmid and recombinant AAV2 constructs

The rat cDNAs of parkin were amplified by PCR. Under the control of the CMV promoter, this fragment was cloned into AAV2-GFP (green fluorescent protein) expression vector. Then AAV2 expression vector, pHelper plasmid, and pCap/rap plasmid were co-transfected into HEK293 cells using Sunbio AAV packing system (Sunbio, Shanghai, China). Cells were harvested after 72h transfection and AAV was purified by iodixanol gradient ultracentrifugation. After several rounds of amplification, AAV titration was finally measured by quantitative PCR using SYBR green technology. The titers of AAV2-GFP-parkin and AAV2-GFP-null were 4.67 × 10^12^ and 5.13 × 10^12^ vector genomes (v.g.)/ml, respectively.

### Injection of AAV2 vectors

The rats were intraperitoneally with a mixture of ketamine (80 mg/kg, Gutian Pharmaceutical Co., Ltd, Fujian, China) and xylazine (5 mg/kg, Sigma-Aldrich, St. Louis, MO) and with topical 0.4% oxybuprocaine hydrochloride (Santen) eye drops. Then rats' pupils were dilated using topical phenylephrine hydrochloride and tropicamide (Santen). A 33-gauge Hamilton syringe (Hamilton Company, Reno, NV, USA) was used to inject 5 μl of AAV2-GFP-parkin or AAV2-GFP-null containing 5 × 10^9^ units of AAV2 virus into the center of the vitreous. Injections were carried out slowly over 1 min, and the syringe was maintained in position for an additional 1 min to minimize loss through the injection tract. Attentions were paid to avoid injury to the lens and retinas.

The rats were examined weekly with a slit lamp for accidental lens puncturing and inflammation. Rat retinas were also examined weekly via an indirect ophthalmoscope under operation microscope. Rats were excluded with punctured lens or damaged retina.

### Tissue preparations

Three days, 1 week, and 2 weeks after first laser treatment, the rats were anesthetized with intraperitoneal injection of 10% chloral hydrate and then were killed by cervical dislocation method. Both eyes were enucleated and postfixed in 4% paraformaldehyde in phosphate-buffered saline (PBS) overnight at 4 °C. After dehydration in graded sucrose solutions (20–30%), retinas were embedded in OCT compounds (Tissue-Tek; Ted Pella Inc, Redding, CA, USA) and stored at −80 °C. For western blot analyses, whole retinas and optic nerves were used immediately or stored at −80 °C until use.

### Retrograde labeling of RGCs

Fluorochrome (FluoroGold 3% diluted in 10% dimethyl sulfoxide; Sigma-Aldrich, St. Louis, MO) was microinjected into the superior colliculi bilaterally of anesthetized rats (2 μl/injection) 4 days before the first laser treatment as described previously^5^. Eyes obtained from the rats 2 weeks after IOP elevation were fixed and processed in flat-mount preparations. Images of full retina were captured with a confocal microscope (Leica SP8). RGCs were counted in the central retina at a distance of 1–2 mm from the optic disc, in the middle retina (2–3 mm from the optic disc), and in the peripheral retina (3–4 mm from the optic disc, Fig. 2a), and the counts were averaged.

### Transmission electron microscopy

Three rats from each group were used in electron microscopic analysis. The optic nerve heads were dissected on ice and immediately fixed via 2.5% glutaraldehyde (Ted Pella, Redding, CA, USA) in 0.1 M phosphate buffer at 4 °C for at least 2 h. After washing in 0.1 M phosphate buffer for 3 times, tissues were fixed with 1% osmic acid at 4 °C for 2 h. Then optic nerves were washed in 0.1 M phosphate buffer for 3 times, dehydrated using an ascending alcohol series, and embedded in epoxy resin. Ultrathin sections on unmyelinated optic nerve were cut and then examined under a transmission electron microscope.

### Western blot analysis

Retinas (*n* = 4 per group) were mixed with RIPA buffer (Beyotime, China) and ultrasonically smashed to get homogenized solutions. Each sample (10 μg) was separated by polyacrylamide gel electrophoresis and electrotransferred onto polyvinylidene difluoride membranes. Membranes were blocked with 5% nonfat dry milk at room temperature for 1 h, incubated with polyclonal rabbit anti-GFAP antibody (1:10,000; Abcam), polyclonal rabbit anti-Parkin (1:1000; Abcam), polyclonal rabbit anti-optineurin (1:200; Abcam), monoclonal rabbit anti-LC3 (1:2000; Abcam), polyclonal rabbit anti-LAMP1 (1:1000; Abcam), and polyclonal rabbit anti-GAPDH (1:2000; Yesen, China) in primary antibody dilution (Beyotime, China) at 4 °C overnight. The membranes were rinsed with 1×TBST (Worthington) several times, incubated with peroxidase-conjugated goat anti-rabbit IgG (1:5000; Jackson), and developed using chemiluminescence detection (SuperSignal West Femto Substrate Trial Kit, Thermo Fisher). Chemiluminescent images were captured using a Kodak Image Station 4000 MM PRO (Carestream, Rochester, NY, USA) and analyzed with Image J (National Institutes of Health).

### Immunohistochemistry analysis

Immunohistochemical staining of 7-μm frozen sections of full-thickness retina was prepared. Three sections per frozen block from each group (*n* = 3 retinas/group) were used for immunohistochemical analysis. Tissue sections were permeabilized with 0.1% Triton X-100 in PBS for 20 min at room temperature and then washed thrice with PBS. Sections were next blocked with 5% bovine serum albumin/PBS for 1 h at room temperature and then with the primary antibodies against monoclonal mouse anti-GFAP antibody (1:200; Abcam), polyclonal rabbit anti-parkin (1:200; Abcam), or polyclonal rabbit anti-optineurin (1:50; Abcam) for 16 h at 4 °C. After several washes, the tissues were incubated with Alexa Fluor 488-conjugated goat IgG secondary antibody (1:200; Life Technologies) for 1 h at room temperature and then washed with PBS. The sections were counterstained with Hoechst 33342 (1 μg/ml; Life Technologies) in PBS. Images were captured by a confocal microscopy (Leica SP8).

### Cultured RGC studies

Rat RGCs were purified, cultured and infected with adenovirus as previously described^16^. The cells were exposed to cell culture medium containing 100 μM glutamate (Sigma-Aldrich) or 100 μM NMDA (Sigma-Aldrich) for 24 h. Cytotoxicity of RGCs was detected by the LDH Cytotoxicity Detection Kit according to the standard protocol (TaKaRa Biotechnology, Dalian, China). Apoptosis of RGCs was assessed by Hoechst 33342 as previously described^16^. Mitochondria were labeled by 200 nM MitoTracker Red (Molecular Probes, M7512; Life Technologies) at 37 °C for 30 min and observed with a confocal microscope (Leica SP8).

### Statistical analysis

Experiments were repeated at least three times. Data are expressed as mean ± SD. One-way analysis of variance and the Bonferroni *t*-test were used to evaluate study results. A *P* < 0.05 was considered statistically significant.
